# Noncoding RNA as a crucial epigenetic modulator in the degeneration of the ligamentum flavum

**DOI:** 10.1038/s12276-024-01348-2

**Published:** 2024-12-02

**Authors:** Yongzhao Zhao, Qian Xiang, Shuo Tian, Zhenquan Wu, Jialiang Lin, Longjie Wang, Zhuoran Sun, Weishi Li

**Affiliations:** 1 Department of Orthopaedics, Peking University Third Hospital, Beijing, China; 2 Beijing Key Laboratory of Spinal Disease Research, Beijing, China; 3 Engineering Research Center of Bone and Joint Precision Medicine, Ministry of Education, Beijing, China

**Keywords:** Senescence, Ligaments

## Abstract

Ligamentum flavum degeneration, including hypertrophy and ossification of the ligamentum flavum, leads to degenerative spinal stenosis in older adults. However, the underlying mechanisms of ligamentum flavum degeneration remain unclear, and therapeutic strategies are limited. Noncoding RNAs include microRNAs, circular RNAs, and long noncoding RNAs. As important epigenetic modifications, noncoding RNAs are involved in the progression of several age-related diseases, including ligamentum flavum degeneration. Previous studies have shown that noncoding RNAs can regulate the osteogenic differentiation and fibrosis of ligamentum flavum cells by regulating the expression of related genes. In this review, we discuss noncoding RNAs and their role in ligamentum flavum degeneration.

## Understanding non-coding RNAs in spinal stenosis

Degenerative Spinal Stenosis (DSS), a common condition in older adults causing numbness and muscle weakness, is often caused by the breakdown of the ligamentum flavum, a spinal structure. Despite DSS’s commonness, the role of non-coding RNAs, molecules that don’t code for proteins but regulate gene activity, in LF breakdown is not well understood. Researchers reviewed the biological functions of ncRNAs in LF breakdown, focusing on microRNAs, circular RNAs, and long non-coding RNAs, aiming to provide new insights. They identified specific ncRNAs contributing to LF degeneration, suggesting their potential as treatment targets. This research could guide future studies towards non-surgical treatments for DSS. The findings reveal that manipulating these ncRNAs could offer new treatment options. This could lead to targeted therapies addressing DSS’s underlying causes, offering hope for less invasive treatments in the future.

This summary was initially drafted using artificial intelligence, then revised and fact-checked by the author.

## Introduction

Degenerative spinal stenosis (DSS) is a very common condition in older adults and constitutes a heavy economic burden for both individuals and society^1,2^. DSS leads to numbness in the trunk and lower limbs, muscle weakness in the lower limbs, and even paraplegia^2,3^. Notably, several degenerative spinal structures can result in DSS, including intervertebral disc degeneration, spondylolysis, facet joint degeneration, and degeneration of ligamentum flavum (LF) tissues^2,3^. LF degeneration is a common cause of DSS. The LF, which is light yellow, connects the lamina of the vertebral bodies, covering the interlaminar space and forming the dorsal border of the spinal canal^4^. In a normal state, the LF is composed of approximately 70% elastin fibers and 30% collagen fibers organized in parallel layers, and its main function is to maintain the mechanical stability of the spine^5^. The two most common types of LF degeneration are hypertrophy of the ligamentum flavum (HLF) and ossification of the ligamentum flavum (OLF). LF degeneration in HLF is characterized by a loss of elastic fibers and increased number of collagen fibers, which further leads to fibrotic thickening and scar tissue formation. HLF is more common in the lumbar spine, and the hypertrophic LF compresses the spinal cord and nerve roots, eventually causing lumbar spinal stenosis^4^. The primary pathology of OLF is the ectopic ossification of LF tissues, which mainly occurs in the thoracic spine^6,7^. In OLF, the ossified LF narrows the thoracic spinal canal and compresses the spinal cord^6,7^. The mainstream therapy for HLF and OLF is surgical decompression because conservative treatment is usually ineffective^2,3^. However, surgical decompression for HLF and OLF is complicated and has a high risk of complications such as injury to the spinal cord or nerve roots, dural tears, and infection^4,7^. Therefore, there is an urgent need to explore the underlying mechanisms of LF degeneration to identify promising therapeutic targets and avoid surgical intervention.

**Fig. 1:**
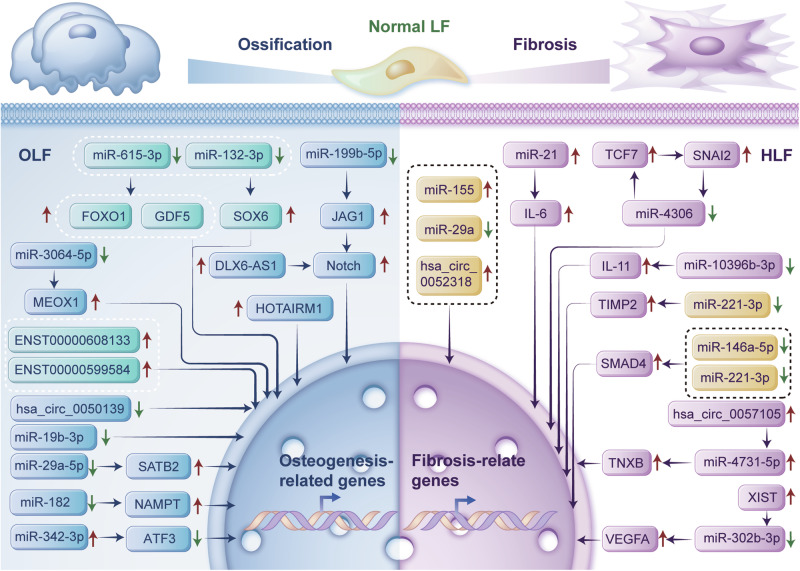
Overview of ncRNAs involved in LF degeneration. This figure illustrates the landscape of function and expression of ncRNAs in LF across different pathological states. LF ligamentum flavum, ncRNA non-coding RNA.

Epigenetic modifications refer to heritable and reversible changes in the function of a gene or genome without altering its DNA sequence^8,9^. Epigenetic modifications include but are not limited to posttranslational modifications, DNA methylation, histone modifications, and noncoding RNA (ncRNA) modulation^9^. Only 2% of transcripts in the human genome can be translated into proteins; however, most (98%) transcripts lack protein-coding ability and are termed ncRNAs^10^. ncRNAs include microRNAs (miRNAs), circular RNAs (circRNAs), and long noncoding RNAs (lncRNAs)^10^. ncRNAs were previously considered genetic “junk”; however, increasing evidence has demonstrated that ncRNAs are crucial in health maintenance and disease progression^11^. ncRNAs are involved in several degenerative musculoskeletal diseases, including osteoarthritis^12^ and intervertebral disc degeneration^13^. Notably, several ncRNAs are associated with the initiation and progression of LF degeneration^14–35^. However, to our knowledge, no specialized systematic review has summarized the critical roles of ncRNAs in LF degeneration. Therefore, in this review, we systematically review the current literature to explore the biological functions of ncRNAs in LF degeneration in order to provide new insights to promote future research and clinical application of ncRNAs in LF degeneration (Fig. 1).

## miRNAs in the degeneration of LF

miRNAs, which constitute the main group of small ncRNAs with average lengths of 19–25 nucleotides, are crucial in the posttranscriptional regulation of gene expression^36,37^. The core function of miRNAs is to induce the translational suppression or degradation of messenger RNAs (mRNAs) through binding to the 3′-untranslated regions (3′-UTRs) of targeted genes, which is called the competing endogenous RNA (ceRNA) mechanism^36,37^. Sixteen miRNAs, including eight HLF-related and eight OLF-related miRNAs, are associated with the progression of HLF and OLF (Table 1).

**Table 1:** miRNAs involved in LF degeneration.

miRNAs	HLF/OLF	Change of expression	Downstream targets	Biological function	References
miR-155	HLF	Increased	NR	Pro-fibrotic	^14^
miR-21	HLF	Increased	IL-6	Pro-fibrotic	^19^
miR-221-3p	HLF	Decreased	TIMP2	Anti-fibrotic	^16^
miR-10396b-3p	HLF	Decreased	IL-11	Anti-fibrotic	^25^
miR-4306	HLF	Decreased	TCF7	Anti-fibrotic	^28^
miR-146a-5p, miR-221-3p	HLF	Decreased	SMAD4	Anti-fibrotic	^30^
microRNA-29a	HLF	Decreased	NR	Anti-fibrotic	^31^
miR-342-3p	OLF	Increased	ATF3	Pro-osteogenic	^33^
miR-132-3p	OLF	Decreased	FOXO1, GDF5, SOX6	Anti-osteogenic	^15^
miR-199b-5p	OLF	Decreased	JAG1/Notch pathway	Anti-osteogenic	^18^
miR-615-3p	OLF	Decreased	FOXO1, GDF5	Anti-osteogenic	^20^
miR-182	OLF	Decreased	NAMPT	Anti-osteogenic	^22^
miR-29a-5p	OLF	Decreased	SATB2	Anti-osteogenic	^23^
miR-19b-3p	OLF	Decreased	NR	Anti-osteogenic	^21^
miR-3064-5p	OLF	Decreased	MEOX1	Anti-osteogenic	^34^

*LF* ligamentum flavum, *HLF* hypertrophy of the ligamentum flavum, *OLF* ossification of the ligamentum flavum, *NR* not reported.

### miRNAs in the HLF

#### miR-155

miR-155 has been previously reported to play important roles in several human diseases. For example, Wang et al. reported that high serum and tumor miR-155 levels are favorable prognostic markers for patients with breast cancer and that the overexpression of miR-155 can promote T-cell influx, delay tumor growth, and sensitize tumors to immune checkpoint blockade therapy in patients with breast cancer^38^. D’Adamo et al. reported that miR-155 suppressed autophagy in chondrocytes by regulating the expression of autophagy proteins, including ULK1, FOXO3, ATG14, ATG5, ATG3, GABARAPL1, and MAP11C3^39^. Chen et al. reported that miR-155 expression was significantly increased in hypertrophic LF tissues compared with normal LF tissues (*p* < 0.01). Moreover, the authors also reported that the miR-155 expression level was positively associated with LF thickness (*p* < 0.01) and the protein levels of fibrosis-related genes (collagen I and III) (*p* < 0.01). Fibroblasts were also transfected with miR-155 mimics or sponge lentiviruses to further explore the biological functions of miR-155 in patients with HLF. The miR-155 mimic significantly increased the mRNA and protein levels of fibrosis-related genes (collagen I and III) in fibroblasts. Conversely, the miR-155 sponge reduced the mRNA and protein expression of fibrosis-related genes (collagen I and III) in fibroblasts. However, further studies on the regulation of miR-155 in patients with HLF have not yet been conducted^14^.

#### miR-21

miR-21 is thought to be involved in many human diseases, such as osteoarthritis^40^ and bone defects^41^. Zhang et al. reported that miR-21 promoted osteoarthritis by targeting GDF5 to decrease GDF5 expression levels and that the knockout of miR-21 could alleviate osteoarthritis in mice^40^. Qi et al. reported that a DNA tetrahedron could facilitate the repair of senescent bone defects by delivering miR-21 to regulate osteogenesis and angiogenesis^41^. Sun et al. explored the role of miR-21 in HLF pathogenesis because it has been reported to participate in the fibrosis of other organs^42,43^. The authors reported that miR-21 expression was significantly increased in hypertrophic LF tissues compared with control LF tissues. Moreover, miR-21 was distinctly associated with LF thickness and fibrosis score in patients with HLF. The overexpression of miR-21 increased the mRNA and protein levels of fibrosis-related genes (collagen I and III) and an inflammation-related gene (IL-6). The authors concluded that miR-21 elevates IL-6 expression, which leads to inflammation, fibrosis, and hypertrophy of LF tissues. However, they did not investigate the mechanism underlying the relationship between miR-21 and IL-6 expression^19^.

#### miR-221-3p

miR-221-3p has been reported to have several biological functions in human diseases, such as intervertebral disc degeneration^44^ and acute myeloid leukemia^45^. Zhang et al. suggested that miR-221-3p in small extracellular vesicles derived from M2 macrophages could inhibit pyroptosis in nucleus pulposus cells through the PTEN/NLRP3 signaling pathway^44^. Li et al. reported that small extracellular vesicles derived from acute myeloid leukemia cells could facilitate leukemogenesis by transferring miR-221-3p to reduce the expression level of GBP2^45^. Xu et al. conducted a miRNA microarray analysis on samples from five patients with and without HLF to screen for potential miRNAs associated with HLF. Eighteen differentially expressed miRNAs were identified, including 15 upregulated (miR-23b, miR-21-5p, miR-202-3p, miR-155, miR-340-5p, miR-374a, miR-146b, miR-29c, miR-223, miR-486, miR-125a, miR-34a, miR-17, miR-148b, and miR-106b) and three downregulated miRNAs (miR-342-5p, miR-27a, and miR-221-3p). Xu et al. selected miR-221-3p from the miRNA microarray results for further investigation. The expression level of miR-221-3p was significantly lower in hypertrophic LF tissues than in normal LF tissues. miR-221-3p overexpression obviously decreased the mRNA levels of fibrosis-related genes (collagen I and III). Moreover, the authors reported that the downregulation of miR-221-3p contributed to HLF by directly targeting the 3’-UTR of TIMP2, a vital protein involved in the breakdown of the extracellular matrix^16^.

#### miR-10396b-3p

Li et al. performed miRNA sequencing analysis on 12 patients with and without HLF. Ninety-four differentially expressed miRNAs were identified, 35 of which were upregulated, and 59 of which were downregulated. The authors selected miR-10396b-3p from the miRNA sequencing analysis for further investigation. miR-10396b-3p expression was distinctly lower in hypertrophic LF tissues than in normal LF tissues. The authors reported that IL-11 is a downstream regulator of miR-10396b-3p and demonstrated that the overexpression of miR-10396b-3p could reduce the mRNA and protein levels of IL-11, which further decreased the expression of fibrotic markers (collagen I and III)^25^.

#### miR-4306

miR-4306 is a well-known miRNA involved in several diseases. Zhao et al. reported that miR-4306 affects the progression of triple-negative breast cancer by directly targeting SIX1/CDC42/VEGFA in triple-negative breast cancer^46^. Wang et al. reported that miR-4306 expression was distinctly decreased in nucleus pulposus tissues; further research indicated that miR-4306 regulated cell proliferation, extracellular matrix synthesis, and autophagy by directly targeting PAK6^47^. Duan et al. reported that TCF7, a member of the T-cell factor/lymphoid enhancer factor transcription factor family, could interact with the SNAI2 promoter to activate the expression of SNAI2, which further promoted the hyperproliferation and fibrosis phenotype of LF cells. Moreover, the authors reported that the level of miR-4036 was significantly lower in hypertrophic LF tissues than in normal LF tissues and that the overexpression of miR-4306 promoted apoptosis but inhibited the viability and fibrosis of LF cells. Notably, the authors reported that miR-4036 was negatively regulated by SNAI2 and could reduce the expression of TCF7 by binding to the 3′-UTR of TCF7 mRNA. In vivo, experiments indicated that knockdown of TCF7 relieved hypertrophy and fibrosis of the LF in mice. The authors demonstrated that the TCF7/SNAI2/miR-4306 feedback loop is critical in HLF pathogenesis^28^.

#### miR-146a-5p and miR-221-3p

miR-146a-5p plays a variety of roles in human diseases. Yang et al. reported that in osteoarthritis, miR-146a-5p relieves the progression of osteoarthritis through the inhibition of SDF-1/CXCR4-induced autophagy in chondrocytes^48^. Rigg et al. reported that tumor-derived extracellular vesicles promoted melanoma brain metastasis via the transfer of miR-146a-5p to regulate the Notch pathway in astrocytes^49^. Ma et al. reported the downregulation of miR-146a-5p and miR-221-3p expression in hypertrophic LF tissues compared with normal LF tissues, and both were negatively correlated with increased LF thickness. The authors reported that umbilical cord mesenchymal stromal cell-derived extracellular vesicles rich in miR-146a-5p and miR-221-3p could relieve hypertrophic LF in in vivo and in vitro assays. Moreover, they demonstrated that miR-146a-5p and miR-221-3p could directly bind to the 3′-UTR regions of SMAD4 mRNA to decrease the SMAD4 expression level, which further blocked the TGF-β/SMAD4 signaling pathway to relieve fibrosis^30^.

#### miR-29a

miR-29a performs multiple biological functions in various diseases. miR-29a can regulate collagen synthesis, activate the NF-κB signaling pathway in tenocytes, and regulate macrophage polarization. Chen et al. reported that lipid nanoparticle-assisted miR-29a delivery via core-shell nanofibers is a promising treatment for tendon injury^50^. Zhao et al. reported that miR-29a inhibited breast tumor initiation by targeting KLF4 in cancer stem cells^51^. Wawrose et al. reported that the level of miR-29a was significantly lower in hypertrophic LF tissues than in normal LF tissues and that miR-29a expression was negatively associated with the mRNA level of collagen I. In in vitro experiments, miR-29a inhibition promoted collagen I mRNA expression. In contrast, the overexpression of miR-29a decreased collagen I mRNA expression. Therefore, the authors concluded that miR-29a is a negative regulator of the pathogenesis of HLF. However, the authors did not investigate the mechanisms underlying the role of miR-29a in the pathogenesis of HLF^31^.

### miRNAs in patients with OLF

#### miR-342-3p

Dysregulated expression of miR-342-3p is involved in many human diseases. Xue et al. reported that miR-342-3p could suppress cell proliferation and migration by directly targeting AGR2 in non-small cell lung cancer^52^. Huang et al. reported that miR-342-3p promoted the osteogenic differentiation of umbilical cord mesenchymal stem cells by reducing SUFU expression^53^. Han et al. selected miR-342-3p on the basis of the results of miRNA sequencing analysis related to OLF^21^. The authors reported that miR-342-3p expression was significantly increased in ossified LF tissues compared with normal LF tissues. Further research revealed that miR-342-3p promoted the osteogenic differentiation of mesenchymal stem cells (MSCs) by directly targeting ATF3. Moreover, the authors reported that the overexpression of miR-342-3p was induced by the demethylation of the Enah/Vasp-Like CpG island^33^.

#### miR-132-3p

miR-132-3p has been reported to play vital roles in several human diseases, especially degenerative diseases. Hu et al. reported that miR-132-3p inhibits osteoblast differentiation by targeting EP300 and could serve as a promising target for the promotion of bone formation^54^. Zhao et al. reported that miR-132-5p could regulate the apoptosis and autophagy of SH-SY5Y cells by decreasing the expression of the target gene ULK1 and that miR-132-5p might be a prospective therapeutic target for Parkinson’s disease^55^. Qu et al. reported that the level of miR-132-3p decreased during the osteogenic differentiation of LF cells. Therefore, inhibiting miR-132-3p promoted the osteogenesis of LF cells; however, the overexpression of miR-132-3p reduced the osteogenesis of LF cells. Furthermore, bioinformatics analysis revealed that FOXO1, GDF5, and SOX6 are potential downstream factors of miR-132-3p, and the authors demonstrated that miR-132-3p inhibited the osteogenesis of LF cells by directly binding to the 3′-UTRs of FOXO1, GDF5 and SOX6^15^.

#### miR-199b-5p

miR-199b-5p is an important epigenetic regulator in the pathogenesis of human diseases. Ruan et al. reported that breast cancer cell-secreted miR-199b-5p promoted brain metastasis by targeting solute carrier transporters to hijack neuron‒astrocyte metabolic coupling^56^. Feng et al. reported that miR-199b-5p was significantly increased during the progression of osteoarthritis and that a reduction in miR-199b-5p could relieve the pathological changes in chondrocytes by targeting FZD6 and GCNT2^57^. Qu et al. selected miR-199b-5p for further investigation because it is reportedly critical in the ossification of the posterior longitudinal ligament and bone marrow stromal cells^58,59^. The authors reported that the expression of miR-199b-5p was significantly decreased during the osteogenesis of LF cells and that the overexpression of miR-199b-5p inhibited the osteogenic differentiation of LF cells. Bioinformatics analysis revealed that miR-199b-5p can directly bind to the 3′-UTR of JAG1, which is a vital Notch ligand and is vital in the Notch signaling pathway^60^. The Notch signaling pathway is crucial for osteogenic differentiation, bone healing, and skeletal development^61^. The authors demonstrated that the overexpression of miR-199b-5p inhibited the osteogenic differentiation of LF cells by targeting JAG1 and modulating the Notch signaling pathway^18^.

#### miR-615-3p

miR-615-3p has multiple biological functions in the pathological and physiological processes of human diseases. For example, miR-199b-5p promoted the chondrogenic differentiation of C3H10T1/2 cells by directly targeting JAG1^62^. In addition, Lin et al. reported that miR-199b-5p could suppress tumor angiogenesis mediated by vascular endothelial cells by reducing ALK1 expression in breast cancer^63^. Yin et al. reported that miR-615-3p was downregulated during the osteogenic differentiation of human osteoblasts, MSCs, and LF cells. In vitro experiments revealed that the overexpression of miR-615-3p inhibited the osteogenic differentiation of LF cells, whereas its knockdown promoted osteogenic differentiation. Moreover, the authors demonstrated that miR-615-3p regulated the osteogenic differentiation of LF cells by directly targeting the 3′-UTRs of FOXO1 and GDF5^20^.

#### miR-182

A variety of studies have explored the important roles of miR-182 in human diseases. Yang et al. reported that miR-182 regulated macrophage polarization and redox reactions to promote repair after ischemic cardiac injury by targeting RASA1^64^. Long et al. reported that exosomal miR-182 derived from bone marrow mesenchymal stem cells contributed to the carfilzomib resistance of multiple myeloma cells by targeting SOX6^65^. Zhang et al. reported that miR-182 expression was significantly decreased, but NAMPT expression was markedly increased in ossified LF tissues compared with normal LF tissues. Furthermore, the authors demonstrated that the overexpression of miR-182 or the inhibition of NAMPT reduced the osteogenic differentiation of LF cells. Notably, bioinformatics analysis and in vitro experiments demonstrated that miR-182 regulated the osteogenic differentiation of LF cells by directly targeting the 3′-UTR of NAMPT mRNA to decrease NAMPT expression^22^.

#### miR-29a-5p

The role of miR-29a-5p in multiple diseases has been widely investigated. Dai et al. reported that miR-29a-5p is a tumor-suppressive miRNA in gliomas and that miR-29a-5p suppresses the proliferation, invasion, and migration of gliomas by directly binding to DHRS4^66^. Wang et al. reported that parathyroid hormone-induced endothelial-to-mesenchymal transition through the miR-29a-5p/GSAP/Notch1 pathway, which further promoted valvular calcification in nonchronic kidney disease^67^. In a study by Feng et al., miR-29a-5p expression was downregulated in ossified LF tissues, whereas SATB2 expression was increased compared with that in normal LF tissues. The authors observed reduced miR-29a-5p expression during the osteogenic differentiation of LF cells, and the overexpression of miR-29a-5p reduced the osteogenic differentiation of LF cells by directly binding to the 3’-UTR of SATB2. Moreover, they reported that SATB2 knockdown significantly reduced SIRT1 levels and Smad3 acetylation. The authors also showed that miR-29a-5p regulated the osteogenic differentiation of LF cells through the SATB2/SIRT1/SMAD3 deacetylation pathway^23^.

#### miR-19b-3p

miR-19b-3p has attracted increasing research attention because of its important biological functions in the maintenance of health. Zhang et al. developed a biomimetic composite hydrogel to facilitate new bone formation via the transfer of miR-19b-3p to regulate WWP1 expression^68^. Chen et al. reported that tumor-derived exosomal miR-19b-3p promoted M2 macrophage polarization by targeting PTPRD, which further inhibited PTPRD-mediated dephosphorylation of STAT3 to promote lung adenocarcinoma metastasis^69^. Han et al. conducted transcriptome sequencing, including miRNA sequencing, on four ossified and four normal LF tissues to explore the changing expression profiles of OLF. The results revealed 28 differentially expressed miRNAs (fold change > 2, *p* < 0.05), including 12 upregulated and 16 downregulated miRNAs. The authors selected miR-19b-3p, a downregulated miRNA identified via miRNA sequencing, for further analyses. They reported that miR-19b-3p was downregulated in ossified LF tissues and human MSCs following osteogenic induction. Therefore, the overexpression of miR-19b-3p decreased the osteogenic differentiation of LF cells. However, the underlying mechanisms have not yet been investigated^21^.

#### miR-3064-5p

miR-3064-5p has been reported to participate in several diseases, such as metainflammation. Luo et al. reported that macrophage exosomes regulate palmitic acid-induced inflammation through the transfer of miR-3064-5p to activate NF-κB signaling by targeting IκBα^70^. Huang et al. reported that, compared with that in normal LF tissues, miR-3064-5p was decreased in ossified LF tissues and gradually decreased in a time-dependent manner during osteogenic differentiation. The overexpression of miR-3064-5p significantly inhibited the osteogenic differentiation of MSCs. MEOX1 is a transcription factor associated with somitic compartment gene expression, embryonic development, and cancer progression^71^. The authors reported that MEOX1 expression was significantly increased in ossified LF tissues and during the osteogenic differentiation of human MSCs. On the basis of bioinformatic analysis and subsequent experimental validation, the authors reported that miR-3064-5p regulated the osteogenic differentiation of MSCs by directly targeting the 3′-UTR of MEOX1^34^.

## Circular RNAs in LF degeneration

CircRNAs are characterized by a single-stranded covalent closed-loop structure without a 5′ cap structure and a 3′ polyadenylated tail^72^. Notably, many circRNAs have been identified through the development of high-throughput RNA sequencing technologies^72^. In recent years, increasing evidence has shown that circRNAs play critical roles in human health and disease by sponging miRNAs or proteins, serving as scaffolds for protein complex formation, and translating them into peptides or proteins^73–75^. CircRNAs have been shown to participate in the pathogenesis of several degenerative diseases of the bone and joints; however, their role in LF degeneration has not been fully reviewed^76,77^. Two circRNAs have been reported to be associated with HLF in LF degeneration, and one circRNA has been reported to contribute to OLF (Table 2).

**Table 2:** circRNAs and lncRNAs involved in LF degeneration.

CircRNA/LncRNA	HLF/OLF	Change of expression	Downstream targets	Biological function	References
hsa_circ_0052318	HLF	Increased	NR	Pro-fibrotic	^24^
hsa_circ_0057105	HLF	Increased	miR-4731-5p/TNXB	Pro-fibrotic	^32^
hsa_circ_0050139	OLF	Increased	NR	Pro-osteogenic	^21^
XIST	HLF	Increased	miR-302b-3p/VEGFA	Proliferation, anti-apoptosis, and fibrosis	^29^
ENST00000608133, ENST00000599584	OLF	Increased	NR	Pro-osteogenic	^21^
HOTAIRM1	OLF	Increased	NF-κB pathway	Pro-osteogenic	^35^
DLX6-AS1	OLF	Increased	Notch pathway	Pro-osteogenic	^27^

*LF* ligamentum flavum, *HLF* hypertrophy of the ligamentum flavum, *OLF* ossification of the ligamentum flavum, *NR* not reported.

### Circular RNAs in patients with HLF

#### hsa_circ_0052318

Chen et al. performed circRNA sequencing analysis on three hypertrophic and three normal LF tissues. HLF tissues were collected from patients with LF hypertrophy at the L4/L5 level, whereas non-HLF tissues were collected from the same patients at the L3/L4 level without evidence of HLF. In total, 2439 differentially expressed circRNAs (fold change ≥ 2, *p* < 0.05) were identified through sequencing analysis. Among the 2439 differentially expressed circRNAs, 1276 were upregulated, and 1163 were downregulated. The authors selected hsa_circ_0052318 for further analysis because it was the largest node in the circRNA‒miRNA coexpression network. In cellular experiments, the authors discovered that the overexpression of hsa_circ_0052318 significantly increased the mRNA and protein levels of collagen I and III. However, the underlying mechanisms have not yet been explored^24^.

#### hsa_circ_0057105

hsa_circ_0057105 is derived from exons 7 to 11 of the PDK1 gene on chromosome 2. Cen et al. reported that hsa_circ_0057105 balanced epithelial‐mesenchymal transition and ferroptosis vulnerability in renal cell carcinoma by sponging miR-577 to regulate COL1A1 and VDAC2 expression^78^. Zhang et al. performed high-throughput second-generation sequencing analysis on five hypertrophic and five normal LF tissues and identified 107 differentially expressed circRNAs, of which 67 were upregulated and 40 were downregulated in hypertrophic tissues compared with normal LF tissues (*p* < 0.05). The authors selected hsa_circ_0057105 for further analysis because it was the most significantly different among the 107 differentially expressed circRNAs. The authors demonstrated that hsa_circ_0057105 expression increased in hypertrophic LF tissues, and cell experiments revealed that hsa_circ_0057105 promoted the viability, proliferation, migration, and fibrosis of LF cells. The authors reported that miR-4731-5p was a potential target of hsa_circ_0057105 and that miR-4731-5p overexpression suppressed the viability, proliferation, migration, and fibrosis of LF cells. TNXB was previously reported to be involved in the osteogenic differentiation of bone marrow MSCs^79^, which was significantly greater in ossified LF tissues than in normal LF tissues. The authors demonstrated that hsa_circ_0057105 promoted the viability, proliferation, migration, and fibrosis of LF cells by increasing TNXB expression by sponging miR-4731. In vivo experiments revealed that miR-4731 knockdown significantly induced fibrosis in LF tissues, whereas the opposite results were observed with hsa_circ_0057105 silencing; moreover, the effect of hsa_circ_0057105 silencing could be partly reversed by miR-4731 knockdown^32^.

### Circular RNAs in patients with OLF

#### hsa_circ_0050139

Han et al. conducted microarray analyses of ncRNAs to explore their potential roles in patients with OLF. In their report, 627 differentially expressed circRNAs were identified with a standard “fold change > 2 and *p* < 0.05”, including 244 upregulated circRNAs and 383 downregulated circRNAs in OLF tissues. Han et al. further validated the role of hsa_circ_0050139 in OLF pathogenesis. They discovered that hsa_circ_0050139 was significantly increased during the osteogenic induction of LF cells and human MSCs. Moreover, osteogenic biomarkers and alkaline phosphatase staining were distinctly decreased after hsa_circ_0050139 was silenced in human MSCs^21^.

## lncRNAs in the degeneration of LF

lncRNAs are a class of transcripts with lengths > 200 nucleotides^80^. Generally, lncRNAs are classified into six categories: sense, antisense, intergenic, intron, bidirectional, and enhancer lncRNAs^80^. lncRNAs can perform biological functions through various mechanisms, such as the regulation of gene expression, chromatin modification, and genomic imprinting. Like circRNAs, the most common biological mechanism of lncRNAs is to regulate the mRNA levels of genes by sponging targeted miRNAs, which is also called the ceRNA hypothesis^80^. Previous studies have demonstrated that lncRNAs are crucial in patients with orthopedic diseases. Five lncRNAs are involved in LF degeneration, including one associated with HLF and four associated with OLF (Table 2).

### lncRNAs in patients with HLF

#### lncRNA X-inactive specific transcript (XIST)

lncRNA XIST, a well-known lncRNA, has been reported to play important roles in several diseases, such as osteoporosis^81^ and multiple cancers^82,83^. Chen et al. reported that lncRNA XIST inhibited osteoblast differentiation and aggravated osteoporosis through Nrf2 hyperactivation by regulating CUL3^81^. Ma et al. reported that lncRNA XIST regulated breast cancer stem cells via the IL-6/STAT3 signaling pathway^83^. With respect to HLF, Cao et al. conducted RNA sequencing on three HLF and three non-HLF tissues and selected lncRNA XIST for further research because it presented the most significant difference in the hub mRNA‒miRNA-lncRNA ceRNA network. In vitro experiments revealed that lncRNA XIST promoted the proliferation, apoptosis, and fibrosis of LF cells by activating autophagy. The authors demonstrated that lncRNA XIST might promote the pathological progression of LF cells by regulating VEGFA expression by sponging miR‑302b‑3p. In vivo, experiments further confirmed that lncRNA XIST knockdown significantly relieved hypertrophy and fibrosis in LF^29^.

### lncRNAs in patients with OLF

#### lncRNA ENST00000608133 and lncRNA ENST00000599584

Han et al. determined the expression profiles of lncRNAs during OLF pathogenesis. In total, 2567 lncRNAs were differentially expressed in OLF tissues (fold change > 2, *P* < 0.05), of which 1817 were upregulated and 750 were downregulated. The authors selected two upregulated lncRNAs (ENST00000608133 and ENST00000599584) for further experiments and demonstrated that the knockdown of ENST00000608133 or ENST00000599584 reduced the osteogenic capacity of human MSCs^21^.

#### lncRNA HOX antisense intergenic RNA myeloid 1 (HOTAIRM1)

Previous studies have shown that lncRNA HOTAIRM1 plays important role in several human diseases. For example, Guo et al. reported that exosome-encapsulated lncRNA HOTAIRM1 promoted airway remodeling in chronic obstructive pulmonary disease by promoting myofibroblast differentiation^84^. Chao et al. reported that lncRNA HOTAIRM1 inhibited cell proliferation and invasion by sponging miR-106a-5p to increase ARHGAP24 expression in ovarian cancer^85^. A study by Ren et al. revealed that lncRNA HOTAIRM1 was upregulated in ossified LF tissues compared with normal LF tissues, according to the GSE106253 microarray data. The authors demonstrated that the expression of lncRNA HOTAIRM1 was increased during osteogenesis in human MSCs and in hFOB1.19, C3H/10T1/2, and MC3T3-E1 cells. Notably, the authors reported that HOTAIRM1 was downregulated during RANKL-induced osteoclast differentiation. Using in vitro experiments, the authors reported that the knockdown of lncRNA HOTAIRM1 inhibited osteoclast differentiation but promoted osteoclast differentiation. The authors demonstrated that lncRNA HOTAIRM1 inhibited RANKL-induced osteoclastogenesis through the NF-κB pathway^35^.

#### lncRNA DLX6 Antisense RNA 14 (DLX6-AS1)

lncRNA DLX6-AS1 performs important biological functions in human diseases. For example, Wang et al. reported that lncRNA DLX6-AS1 promoted myocardial ischemia-reperfusion injury via the miR-204-5p/FBXW7 axis^86^. Guo et al. reported that lncRNA DLX6-AS1 promoted cell proliferation, invasion, migration, and epithelial-to-mesenchymal transition by modulating the Wnt/β-catenin signaling pathway in bladder cancer^87^. Zhang et al. reported that the lncRNA DLX6-AS1, also known as lnc-EVF2, was expressed at higher levels in LF samples than in normal LF tissues, according to the microarray data of the GSE106253 dataset. The authors also reported that lncRNA DLX6-AS1 was gradually upregulated during osteogenic induction at various time points in MC3T3-E1, C3H10T1/2, and mouse MSCs. They demonstrated that the overexpression of lncRNA DLX6-AS1 promoted osteogenic differentiation by regulating the Notch signaling pathway^27^.

In conclusion, DSS due to LF degeneration has become the main contributor to low back pain, which places a heavy economic burden on society and individuals^2,3^. However, there are currently no reliable biomarkers for predicting or treating LF degeneration. As critical components of epigenetic modifications, ncRNAs, including miRNAs, circRNAs, and lncRNAs, are crucial in the onset and development of LF degeneration. However, there is still a long way to go before clinical translation can be achieved for ncRNAs in LF degeneration. First, while all of these ncRNAs have been proven to be effective in cell or animal experiments, none have been verified in humans. Second, more detailed and accurate mechanisms of these ncRNAs in LF degeneration have not been reported, such as why a given ncRNA is upregulated or downregulated. Third, the expression levels of these ncRNAs have not been assessed in the blood samples of patients, which limits the application of ncRNAs as diagnostic markers or monitoring indicators for LF degeneration. Fourth, ncRNAs can perform their biological functions through several molecular mechanisms, such as serving as protein scaffolds and translating them into peptides. However, all currently published studies have focused only on the ceRNA mechanism of ncRNAs in LF degeneration. Therefore, future studies should be conducted to address these concerns. These efforts will benefit the management of patients with LF degeneration through the application of more accurate, effective, and tailored ncRNA therapies.
